# Allogeneic stem cell transplantation for peripheral T cell lymphomas: a retrospective study in 285 patients from the Société Francophone de Greffe de Moelle et de Thérapie Cellulaire (SFGM-TC)

**DOI:** 10.1186/s13045-020-00892-4

**Published:** 2020-05-19

**Authors:** Anne-Claire Mamez, Axelle Dupont, Didier Blaise, Patrice Chevallier, Edouard Forcade, Patrice Ceballos, Mohamad Mohty, Felipe Suarez, Yves Beguin, Regis Peffault De Latour, Marie-Thérèse Rubio, Olivier Tournilhac, Stéphanie Nguyen

**Affiliations:** 1 Hematology Department, Hôpitaux Universitaires de Genève, 4 rue Gabrielle-Perret- Gentil, 1205 Geneva, Switzerland; 2 APHP Department of Biostatistics and Medical Information, Hôpital Saint-Louis; ECSTRA Team, CRESS UMR 1153, INSERM, Paris Diderot University, Paris, France; 3 Institut Paoli-Calmettes, Aix Marseille Université, CNRS, INSERM, CRCM, Marseille, France; 4 Hematology Department, CHU Hôtel-Dieu, Nantes, France; 5 Service d’Hématologie Clinique et Thérapie Cellulaire, CHU Bordeaux, Bordeaux, France; 6 Hematology Department, Hôpital St Eloi, Montpellier, France; 7 Hematology Department, Saint Antoine Hospital, Paris, France; 8 Hematology Department, Hôpital Necker, Paris, France; 9 Department of Medicine, Division of Hematology, University of Liège, Liège, Belgium; 10 Bone Marrow Transplant Unit, Department of Hematology, Saint-Louis Hospital, University Paris-Diderot, Paris, France; 11 Hematology Department, CHRU Nancy, Hôpital Barbois, Vandœuvre-les, Nancy, France; 12 Hematology Department, CHU Clermont-Ferrand, Clermont-Ferrand, France; 13 Department of Hematology, Hôpital Pitié Salpêtrière, Paris, France

**Keywords:** Allogeneic stem cell transplantation, Peripheral T cell lymphoma, Retrospective analysis

## Abstract

**Background:**

Peripheral T cell lymphomas form a heterogeneous group with a usually dismal prognostic. The place of allogeneic stem cell transplantation to treat PTCL is debated.

**Methods:**

We retrospectively analyzed the overall survival (OS), event-free survival (EFS), relapse, and transplant-related mortality (TRM) and associated variables in 285 adults with non-primary cutaneous PTCL (PCTL-NOS (39%), angioimmunoblastic T cell lymphomas (29%), anaplastic T cell lymphomas (15%), and other subtypes (17%)), who received alloSCT in 34 centers between 2006 and 2014.

**Results:**

AlloSCT was given as part of front-line therapy (*n* = 138) to 93 patients in first complete response (CR) and 45 in first partial response (PR), and of salvage therapy (*n* = 147) to 116 patients for second or more CR/PR and 31 for progressive disease. Reduced-intensity conditioning (RIC) was given to 172 patients (62%), while 107 (38%) received myeloablative conditioning (MAC). The median follow-up was 72.4 months. The 2- and 4-year OS were 65% and 59%, respectively, and the cumulative incidence of relapse was 18% after 1 year and 19% after 2 years. TRM was 21% at 1 year, 24% after 2 years, and 28% after 4 years. In multivariate analysis, grade III–IV acute GvHD (HR = 2.57, 95% CI 1.53–4.31; *p* = 0.00036), low Karnofsky score < 80% (HR = 5.14, 95% CI 2.02–13.06; *p* = 0.00058), and progressive disease status before transplant (HR = 2.21, 95% CI 1.25–3.89; *p =* 0.0062) were significantly associated with a reduced OS.

**Conclusions:**

The data demonstrate in the largest retrospective cohort of non-cutaneous PTCL so far reported that alloSCT after RIC or MAC is an effective strategy, even in chemoresistant patients.

## Background

Peripheral T cell lymphomas (PTCL) form a heterogeneous group of rare lymphomas [1], with a usually dismal prognostic.

For patients treated with CHOP-like regimen, the overall response rate is about 50% [2] and the long-term outcome remains poor, with a 3-year event-free survival (EFS) below 50% for PTCL, except for ALK+ anaplastic large cell lymphoma (ALCL) [3]. For relapsed or refractory patients, a study including 153 PTCL patients reported a poor outcome in the absence of hematopoietic transplantation, even when receiving chemotherapy salvage regimen, with a median OS and PFS of 13.7 months and 5 months, respectively [4].

Because of better knowledge of nosology and biology of lymphomas, new targeted drugs have been developed, including pralatrexate and romidepsin, crizotinib for ALK-positive ALCL, and brentuximab vedotin for CD30-positive PTCL with promising response rate, although the impact on long-term disease control remains unclear [5, 6]. For eligible patients with chemosensitive disease, the use of high-dose chemotherapy followed by autologous SCT (autoSCT) has been recommended as first-line consolidation therapy [7, 8], but the efficacy is still a matter of debate [9, 10]. The incidence of relapse after autoSCT remains high, and 30 to 40% of patients experience early relapse before any chance of receiving consolidation therapy [11, 12]. The development of CAR-T cells has revolutionized the treatment of B cell lymphoma. However, targeting malignant T cells with immunotherapy is more complex and challenging [13].

In this context, the question of alloSCT for PTCL (5) remains relevant. Graft-versus-lymphoma (GVL) effect has been described in PTCL [14]. Indeed, survival has been shown to plateau after alloSCT [15], even after RIC [16]. There is also a potential effect of donor lymphocyte infusion (DLI) in post-transplant relapse [17], and Kanakry et al. reported a reduced incidence of relapse (17% compared to 66%, *p* = 0.04) in patients who developed GvHD [18]. However, because of high TRM, recommending alloSCT for PTCL remains a matter of debate, and current guidelines limit its use for relapsed or refractory patients [7, 8].

With the goal to analyze the outcome of alloSCT in a large number of patients with non-primary cutaneous PTCL, we performed a retrospective analysis in 285 patients.

## Methods

### Study design, inclusion criteria, data collection, and definitions

This study was based on the SFGM-TC registry. Patients with PTCL who underwent alloSCT in 32 centers between October 2006 and January 2014 were included. Patients with primary cutaneous T cell lymphoma younger than 15 years of age were excluded. The study was approved by the SFGM-TC scientific council. Informed consent was obtained from the patients in accordance with the Declaration of Helsinki.

Complete response (CR) was defined as the complete disappearance of clinical, radiological, and laboratory evidence of disease. Partial response (PR) was defined as a 50% or greater reduction in tumor mass. Progressive disease (PD) was defined as a > 25% increase in tumor mass. Relapse was defined as the recurrence of clinical or radiological signs of disease. Acute and chronic GvHD was graded according to international criteria [19].

### Statistical analysis

Different outcomes were used, such as death, EFS, relapse, and TRM. The graft versus host disease-free relapse-free survival (GRFS) was defined as the time when the first event among death, progression/relapse, grade 3–4 acute GvHD, or extensive chronic GvHD occurred after alloSCT. Survival curves were estimated using the Kaplan-Meier product limit estimator. Competing risk survival analysis methods were applied to estimate the cumulative incidence of relapse (CIR) (death as a competing risk) over time from alloSCT. Factors associated with OS and therapy-related mortality (TRM) were analyzed using Cox proportional hazard models. The proportional hazard assumption was checked by examination of the scaled Schoenfeld residuals. Occurrence of acute GvHD or chronic GvHD was treated as a time-dependent co-variable. For relapse, associations were analyzed with the Fine and Gray models. The impact of chronic GvHD on relapse was studied with landmark analysis at different times.

For each outcome, univariate analyses were first carried out, followed by multivariate analyses that included all factors with a *p* value < 0.1 in the univariate analyses. If needed, factors were then sequentially removed from the adjusted model based on AIC criteria. To explore the impact of the conditioning regimen on OS, a propensity score was constructed, excluding patients who could not receive MAC, i.e., patients older than 50 years, with a Karnofsky score under 70, or who had previously received autoSCT.

## Results

### Patients’ and treatment-related characteristics at transplant

Patients’ characteristics are summarized in Table 1 and transplant features in Table 2. Median age at transplantation was 49.5 years old. Histological subtypes were PTCL-NOS (*n* = 110), angioimmunoblastic T lymphomas (AITL, *n* = 83), ALCL (*n* = 43), NK/T lymphoma nasal type (*n* = 16), HSTL (*n* = 12), EATL (*n* = 3), T large granular lymphocytic leukemia (T-LGL, *n* = 1), and NK leukemia (*n* = 1).

**Table 1:** Patients’ characteristics

		*N*/med [min–max]	Percentage
Patients		285	
Sex	Male	191	67
Age at transplant (yo)	Median	49.5 [16–69]	
	20–40	87	31
	41–60	157	55
	> 60	41	14
Histological subtype	NOS	110	39
	AITL	83	29
	ALCL ALK+	21	7
	ALCL ALK−	20	7
	ALCL ALK unknown	2	< 1
	ATLL	16	6
	NK/T	16	6
	HSTL	12	4
	EATL	3	1
	LGL	1	< 1
	NK leukemia	1	< 1
Stage at diagnosis	I–II	30	15
	III–IV	172	85
	Missing data	83	
Place of alloSCT	Front-line consolidation	138	48
	-CR1	93	33
	-PR1	45	15
	Second-line consolidation	116	41
	-CR2	72	25
	-CR > 2	13	5
	-PR2	25	9
	-PR > 2	6	2
	Progressive disease	31	11
Previous autoSCT	No previous autoSCT	192	67
	Yes (patient in relapse after autoSCT)	66	23
	Yes (tandem auto/alloSCT)	27	9
Karnofsky score at alloSCT	100%	93	35
	90–80%	156	59
	≤ 70%	15	6
	Missing data	21	

*med* median, *min* minimum, *max* maximum, *yo* years old, *NOS* not otherwise specified, *AITL* angioimmunoblastic T lymphoma, *ALCL* anaplastic large cell lymphoma, *ALK+/−* with/without anaplastic lymphoma kinase mutation, *ATLL* adult T cell leukemia/lymphoma, *NK/T* NK/T cell lymphoma, *HSTL* hepatosplenic T cell lymphoma, *EATL* enteropathy-associated T cell lymphoma, *LGL* large granular lymphocyte leukemia, *CR* complete remission, *PR* partial remission, *SCT* stem cell transplantation

**Table 2:** Transplantation features

			Number	Percentage
Patients			285	
Disease status at transplant	Complete response	Total CR	178	62
		CR1	93	
		CR ≥ 2	85	
	Partial response	Total PR	76	27
		PR1	45	
		PR ≥ 2	31	
	Progressive disease		31	11
Time from diagnosis to transplant		< 12 months	149	52
Conditioning regimen	RIC		174	62
	MAC	Total MAC	107	38
		TBI-based MAC	67	
	Missing data		4	
Graft source	PBSC		203	71
	Bone marrow		49	17
	CB		33	12
Sex of donor/recipient	F/M		74	27
CMV serostatus	Neg/neg		92	32
HLA matching	Sibling identical		128	45
	Matched unrelated donor (10/10)		104	36
	Mismatched unrelated donor		13	5
	Cord blood		33	12
	Haploidentical		7	2
T depletion	In vivo T depletion (ATG)		142	50
	Ex vivo T depletion		4	1

*CR* complete remission, *PR* partial remission, *RIC* reduced-intensity conditioning regimen, *MAC* myeloablative conditioning regimen, *PBSC* peripheral blood stem cells, *CB* cord blood, *F* female, *M* male, *neg* negative, *ATG* globulin anti-thymocytes

The median number of treatment lines before transplant was 2 (1, 29%; 2, 36%; 3, 26%; > 3, 9%). Induction chemotherapy was mainly based on a CHOP-like regimen.

AlloSCT was performed in 138 patients as part of front-line therapy (93 in first CR (CR1), 45 in first PR (PR1)), while 147 patients were allografted either as salvage therapy for progressive disease (PD; *n* = 31) or as second-line consolidation after CR or PR for relapse after chemo (*n* = 56) or after autoSCT (*n* = 60).

For the 66 patients in the cohort (23%) who experienced relapse after autoSCT, the median time between auto- and alloSCT was 19 months (6–105 months).

At the time of alloSCT, 178 patients (62%) were in CR, 76 (27%) were in PR, and 31 (11%) had PD. The median time from diagnosis to alloSCT was 12.6 months.

The majority (*n* = 174; 62%) of patients received RIC regimens. Compared to the RIC group, patients who received a MAC regimen were significantly younger, had less frequently undergone a previous autoSCT, and had a shorter time from diagnosis to alloSCT and fewer CR at time of transplantation (see appendices).

Twenty-seven patients were treated with a tandem auto/alloSCT (4 MAC, 23 RIC) with a median time between transplants of 98 days; before transplant, 22/27 were in CR, 4/27 were in PR, and one patient had PD.

GvHD prophylaxis was mainly based on cyclosporin +/− mycophenolate mofetil or methotrexate.

### Post-alloSCT outcomes

Acute GvHD (grades II–IV) occurred in 30% of the patients (grades III–IV = 14.7%). One third (*n* = 106) developed chronic GvHD, which was extensive in 14.8% of cases.

Sixty-five patients experienced post-alloSCT lymphoma relapse (see appendices). Three patients who received consolidation treatment (DLI, *n* = 2; radiotherapy, *n* = 1) for persistent post-transplant PR were in CR at the last follow-up. One patient received a second alloSCT from the same donor because of graft rejection.

One hundred and eighteen patients died during the follow-up period. The main causes of death were GvHD (8%; *n* = 24) and infections (10%; *n* = 30), whereas 41 patients died of lymphoma relapse (14%).

### Overall survival, event-free survival, relapse/progression, transplant-related mortality, and graft versus host disease-free relapse-free survival

**Fig. 1:**
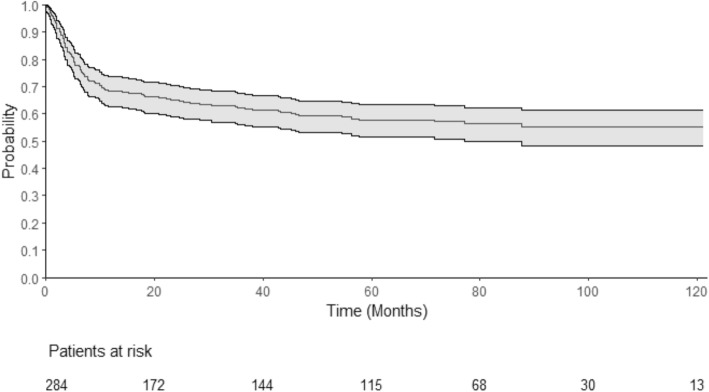
Overall survival. This Kaplan-Meier curve represents the probability of overall survival for the all cohort, from the time of alloSCT (stem cell transplantation) to death or loss to follow-up. The continuous line represents the survival curve; the 2 dotted lines represent the 95% CI. Time is represented in months on the horizontal axis. Below the *x*-axis, the remaining patients at risk are detailed. OS at 1 year was 68% (95% CI 0.63–0.74) and was 65% (95% CI 0.59–0.7) at 2 years. OS at 4 years was 59% (95% CI 0.53–0.65)

**Fig. 2:**
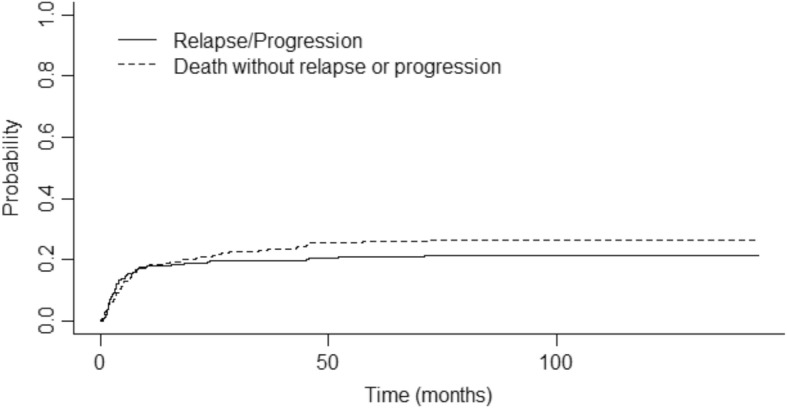
Cumulative incidence of relapse and non-relapse mortality. This curve represents the probability of cumulative incidence of lymphoma relapse (continuous line) and the probability of death without relapse/ progression (dotted line) from the time of transplant. Time is represented in months on the horizontal axis. Cumulative incidence for relapse at 1 year was 0.18 (95% CI 0.13–0.23) and at 2 years 0.19 (95% CI 0.14–0.24). Cumulative incidence for death without relapse at 1 year was 0.18 (95% CI 0.14–0.23) and 0.21 at 2 years (95% CI 0.16–0.26)

The median follow-up was 72.4 months (95% CI 69.4–79.5). One-year, 2-year, and 4-year OS were 68% (95% CI 0.63–0.74), 65% (95% CI 0.59–0.7), and 59% (95% CI 0.53–0.65), respectively (Fig. 1). One-year, 2-year, and 4-year EFS were 64% (95% CI 0.58–0.7), 60% (95% CI 0.54–0.66), and 54% (95% CI 0.48–0.61), respectively. The cumulative incidence of relapse (CIR) was 18% after 1 year (95% CI 0.13–0.23) and 19% after 2 years (95% CI 0.14–0.24) (Fig. 2). The median time from transplant to relapse was 97 days, and only 10% of the relapse occurred after the first-year post-transplant. TRM was 21% at 1 year (95% CI 0.17–0.27), 24% after 2 years (95% CI 0.3–0.19), and 28% (95% CI 0.34–0.23) after 4 years. GRFS at 1 year, 2 years, and 4 years was respectively 49% (95% CI 0.43–0.55), 46% (95% CI 0.40–0.52), and 43% (95% CI 0.37–0.49).

### Factors associated with outcome

Univariate and multivariate analyses for OS are summarized in Table 3. In multivariate analyses, grade III–IV acute GvHD (HR = 2.57, 95% CI 1.53–4.31; *p* = 0.00036), low Karnofsky score < 80% (HR = 5.14, 95% CI 2.02–13.06; *p* = 0.00058), and PD versus CR before transplant (HR = 2.21, 95% CI 1.25–3.89; *p =* 0.0062) were significantly associated with the 5-year OS. The main factors associated with TRM were number of lines of treatment ≤ 2 (HR = 0.59, 95% CI 0.35–0.99; *p* = 0.047) and low Karnofsky score < 80% (HR = 3.43, 95% CI 1.09–10.7; *p* = 0.034) (see appendices).

**Table 3:** Univariable and multivariable analyses for overall survival

Univariable analysis for 5-year OS		HR (95% CI)	*p* value
Age at transplant		1.00 (0.99–1.02)	0.68
Histology subtypes	AITL	1.00	0.30
	ALCL*	1.03 (0.56–1.92)	
	ATLL	1.62 (0.77–3.43)	
	NOS	0.79 (0.48–1.3)	
	NK/T nasal	1.93 (0.92–4.07)	
	HSTL	0.79 (0.24–2.6)	
	Other subtypes	1.32 (0.32–5.54)	
Time from diagnostic to alloSCT > 12 months		0.9 (0.6–1.33)	0.59
Number of lines of treatment ≤ 2		0.71 (0.47–1.08)	0.11
Karnofsky score at transplant (%)	100	1.00	0.001
	80–90	2.08 (1.27–3.4)	
	< 80	4.00 (1.7–9.41)	
Disease status at transplant	CR	1.00	0.011
	PR	0.83 (0.51–1.37)	
	PD	2.13 (1.27–3.57)	
HLA-matched donor		0.71 (0.45–1.12)	0.16
Conditioning regimen	MAC	1.00	0.86
	RIC	0.96 (0.64–1.45)	
CMV status (D/R)	Neg/neg	1.00	0.89
	Others	0.97 (0.63–1.49)	
Mismatch sex (F/H vs others)		1.39 (0.91–2.12)	0.14
Source of stem cells	BM	1.00	0.022
	Cord blood	1.44 (0.76–2.73)	
	PBSC	0.69 (0.41–1.14)	
Acute GvHD (grades 3–4)		2.69 (1.67–4.33)	0.0002
Chronic GvHD		1.22 (0.72–2.06)	0.47
Multivariable analysis for 5-year OS		HR (95% CI)	*p* value
Acute GvHD (grade 3-4)		2.57 (1.53–4.31)	0.00036
Karnofsky score (%)	90–80 vs 100	2.07 (1.24–3.44)	0.0053
	< 80 vs 100	5.14 (2.02–13.06)	0.00058
Disease status before transplant	PR vs CR	0.72 (0.42–1.24)	0.24
	PD vs CR	2.21 (1.25–3.89)	0.0062
Stem cell source	Cord blood vs BM	1.78 (0.90–3.51)	0.10
	PBSC vs BM	0.85 (0.50–1.45)	0.54

*OS* overall survival, *HR* hazard ratio, *CI* confidence interval, *AITL* angioimmunoblastic T lymphoma, *ALCL* anaplastic large cell lymphoma, *ALK+/−* with/without anaplastic lymphoma kinase mutation, *ATLL* adult T cell leukemia/lymphoma, *NK/T* NK/T cell lymphoma, *HSTL* hepatosplenic T cell lymphoma, *SCT* stem cell transplantation, *CR* complete remission, *PR* partial remission, *PD* progressive disease, *MAC* myeloablative conditioning regimen, *RIC* reduced-intensity conditioning regimen, *neg* negative, *BM* bone marrow, *PBSC* peripheral blood stem cells, *F* female, *M* male, *GvHD* graft versus host disease, *D/R* donor/recipient

*Polled data of ALK+ and ALK− ALCL

Outcomes according to the histologic subtypes are detailed in Table 4. Outcomes according to the timing of alloSCT (front-line or second-line treatment) and disease status at transplant are detailed in Table 5. Of note, 31 patients with PD underwent alloSCT (RIC, *n* = 22; MAC, *n* = 9), and among them, 7 had primary refractory disease and received alloSCT as first-line salvage treatment. In this subgroup, 47% (*n* = 15) reached CR after transplant.

**Table 4:** Survival analysis according to histological lymphoma subtypes

	Number	OS, % (95% CI)		EFS, % (95% CI)		TRM, % (95% CI)		CI relapse/progression, % (95% CI)	
		1 year	2 years	1 year	2 years	1 year	2 years	1 year	2 years
T-NOS	110	72% (0.61–0.79)	68% (0.58–0.76)	66% (0.58–0.76)	61% (0.52–0.71)	16% (0.1–0.25)	20% (0.13–0.29)	19% (0.12–0.27)	21% (0.13–0.29)
AITL	83	73% (0.62–0.81)	67% (0.56–0.77)	71% (0.61–0.82)	64% (0.54–0.76)	23% (0.15–0.35)	28% (0.28–0.39)	10% (0.03–0.17)	12% (0.04–0.19)
ALCL ALK+	21	81% (0.57–0.92)	81% (0.57–0.92)	71% (0.54–0.94)	71% (0.54–0.94)	5% (0.01–0.32)	5% (0.01–0.32)	24% (0.05–0.43)	24% (0.05–0.43)
ALCL ALK−	20	55% (0.28–0.72)	50% (0.28–0.68)	52% (0.35–0.79)	52% (0.35–0.79)	34% (0.18–0.58)	34% (0.18–0.58)	14% (0–0.3)	14% (0–0.3)
NK/T	16	50% (0.25–0.71)	50% (0.25–0.71)	44% (0.25–0.76)	44% (0.25–0.76)	29% (0.12–0.61)	29% (0.12–0.61)	–	–
ATLL	16	56% (0.29–0.76)	56% (0.29–0.76)	38% (0.2–0.71)	38% (0.2–0.71)	25% (0.09–0.59)	25% (0.09–0.59)	44% (0.18–0.69)	44% (0.18–0.69)
HSTL	12	58% (0.27–0.8)	58% (0.27–0.8)	64% (0.41–0.99)	64% (0.41–0.99)	42% (0.2–0.73)	42% (0.2–0.73)	–	–
EATL	3	67% (0.05–0.95)	67% (0.05–0.95)	67% (0.3–1)	67% ( 0.3–1)	0	0	–	–

*OS* overall survival, *EFS* event-free survival, *TRM* toxic-related mortality, *CI* confidence interval, *NOS* not otherwise specified, *AITL* angioimmunoblastic T lymphoma, *ALCL* anaplastic large cell lymphoma, *ALK+/−* with/without anaplastic lymphoma kinase mutation, *NK/T* NK/T cell lymphoma, *ATLL* adult T cell leukemia/lymphoma, *HSTL* hepatosplenic T cell lymphoma, *EATL* enteropathy-associated T cell lymphoma

**Table 5:** Outcomes for all the group and according the timing of alloSCT (front-line, second-line treatment, or progressive disease)

	Number	Overall survival, % (95% CI)		Cumulative incidence of relapse, % (95% CI)	TRM, % (95% CI)		GRFS, % (95% CI)
		2-year OS	4-year OS	At 2 years	2-year TRM	4-year TRM	2-year GRFS
All group	285	65% (0.59–0.7)	59% (0.53–0.65)	19% (0.14–0.24)	24% (0.3–0.19)	28% (0.34–0.23)	46% (0.4–0.52)
Front-line alloSCT (CR1 + PR1)	138	66% (0.58.0.74)	63% (0.53–0.7)	19% (0.12–0.25)	23% (0.16–0.31)	24% (0.17–0.32)	48% (0.39–0.56)
CR1	93	71% (0.6–0.79)	62% (0.51–0.71)	14% (0.13–0–51)	26% (0.53–0.74)	27% (0.38–0.19)	48% (0.37–0.58)
Second-line alloSCT (CR ≥ 2 or PR ≥ 2)	116	66% (0.56–0.74)	61% (0.51–0.7)	17% (0.1–0.24)	25% (0.18–0.35)	30% (0.22–0.4)	45% (0.36–0.54)
Progressive disease	31	55% (0.36–0.7)	37% (0.2–0.54)	32% (0.13–0.52)	24% (0.46–0.12)	40% (0.63–0.23)	30% (0.19–0.56)

*SCT* stem cell transplantation, *TRM* toxic-related mortality, *OS* overall survival, *CR* complete remission, *PR* partial remission, *GRFS* graft versus host disease-free relapse-free survival

**Fig. 3:**
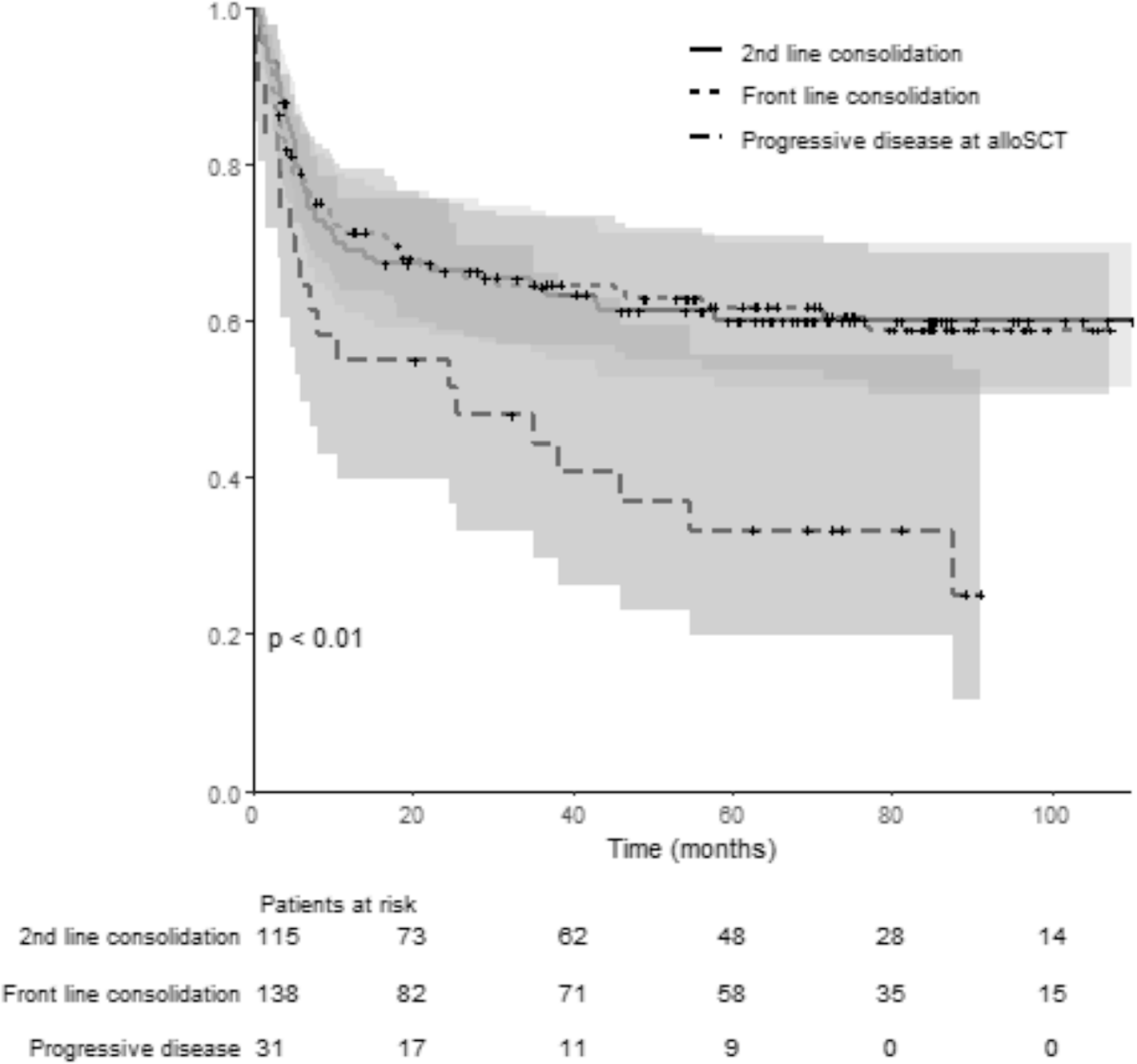
Overall survival for patients allotransplanted in front-line consolidation (CR1/PR1), second-line consolidation (CR2 or more /PR2 or more), and/or refractory/progressive disease. This curve represent the probability of overall survival from the time of alloSCT (stem cell transplantation) for patients allotransplanted in front-line consolidation (pointed line, CR1/PR1), second-line consolidation (continuous line, CR2 or more/PR2 or more), and/or refractory/progressive disease (dotted line). The gray zones represent the 95% CI for each subgroup. Time is represented in months on the horizontal axis. Below the *x*-axis, the number of patients at risk for each group is detailed. The *p* value (log rank test) is significant (*p* < 0.01) comparing OS among groups

**Fig. 4:**
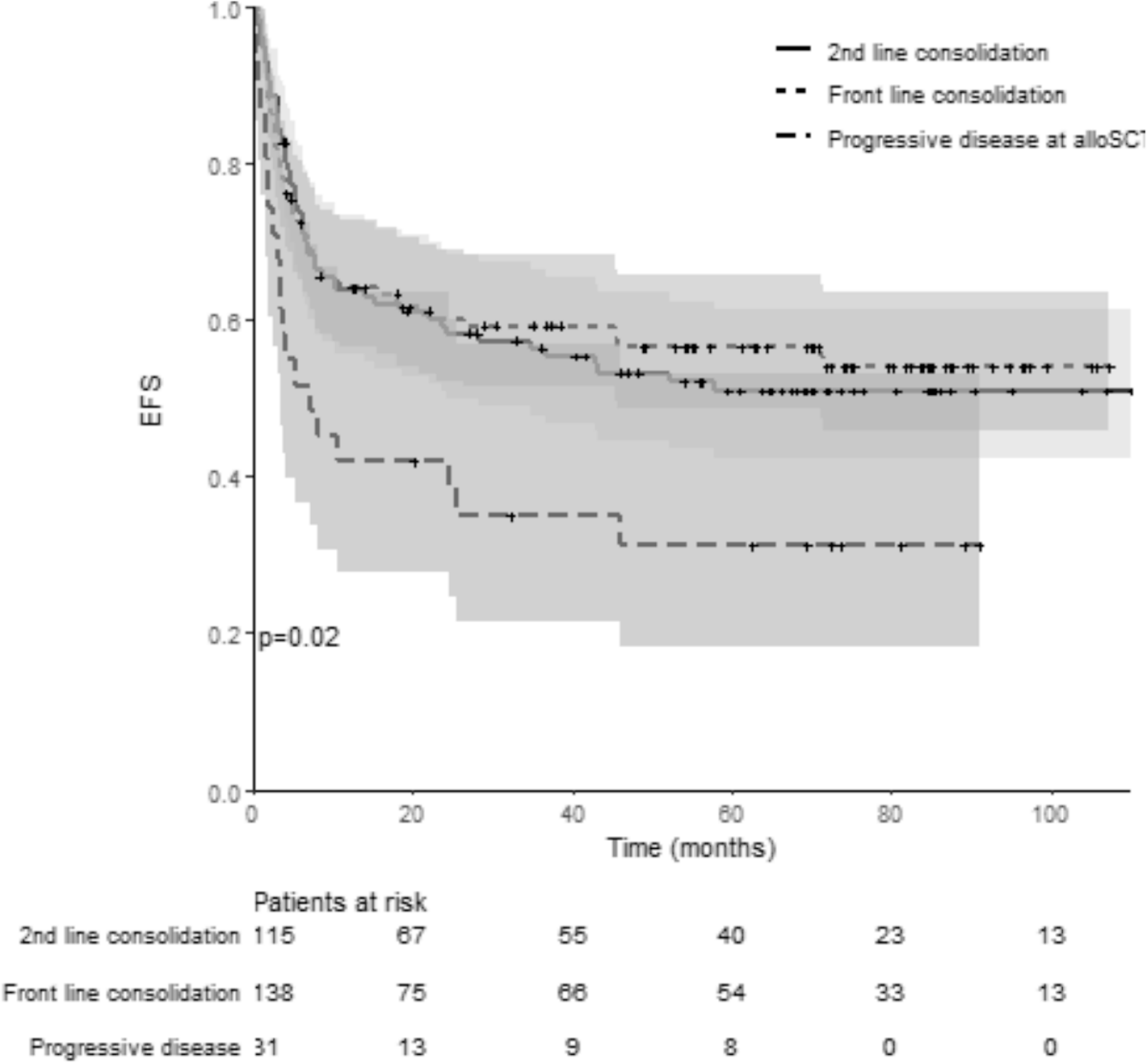
Event-free survival for patients allotransplanted in front-line consolidation (CR1/PR1), second-line consolidation (CR2 or more /PR2 or more), and/or refractory/progressive disease. This Kaplan-Meier curve represents the probability of event-free survival from the time of alloSCT (stem cell transplantation) for patients allotransplanted in front-line consolidation (pointed line, CR1/PR1), second-line consolidation (continuous line, CR2 or more /PR2 or more), and/or refractory/progressive disease (dotted line). The gray zones represent the 95% CI for each subgroup. Time is represented in months on the horizontal axis. Below the *x*-axis, the number of patients at risk for each group is detailed. The *p* value (log rank test) is significant (*p* = 0.02) comparing EFS among groups

OS and EFS according to the disease status before transplant (CR1/PR1 vs CR ≥ 2/PR ≥ 2 vs PD) are illustrated in Figs. 3 and 4. The *p* value (log rank test) is significant (*p* < 0.01) comparing OS (*p* < 0.01) and EFS (*p* 0.02) among groups. No differences were found among the three groups of patients for GRFS (log rank test: *p* = 0.08).

### Outcomes according to conditioning regimen intensity

The 2-year CIR was 22% in the MAC group and 17% in the RIC group. The 2-year TRM was 21% (95% CI 0.12–0.29) in the MAC group and 24% (95% CI 0.17–0.31) in the RIC group. To note, because of a great heterogeneity in terms of TBI dose associated with various conditioning regimen, we chose to perform the comparison between MAC and RIC.

Significance was tested by constructing a propensity score after excluding patients who could not receive MAC. We did not find any significant difference between MAC and RIC for OS (*p* = 0.5), EFS (*p* = 0.55), TRM (*p* = 0.09), or relapse risk (*p* = 0.32).

### Outcome according to chronic GvHD

One hundred and six developed chronic GvHD, and only 11/106 experienced lymphoma relapse after alloSCT. In this subgroup of patients with chronic GvHD, the 2-year OS and EFS after chronic GVHD diagnosis were 78% (95% CI 0.7–0.87) and 68% (95% CI 0.6–0.78), respectively. After landmark analysis, no significant difference on cumulative incidence of relapse was found for patients who developed chronic GvHD compared to the ones who did not.

## Discussion

To the best of our knowledge, this series, including 285 patients with non-primary cutaneous PTCL treated with alloSCT, is the largest to be reported so far. The study shows promising results for OS and EFS (65% and 60% at 2 years, 59% and 54% at 4 years) despite the heterogeneity of the population and the retrospective nature of the study. A plateau was reached after 1 year for OS and EFS with fewer relapses occurring, although relapses were responsible for the death of 14% of the patients overall. Pre-transplant disease status (PD vs CR) and receiving more than two lines of treatment were significantly associated with OS.

One of the major issues when considering alloSCT for PTCL patients, whose median age at diagnosis is 50 to 60 years old, is the TRM, which was high, and accounted for 25% of the deaths, even in patients transplanted as front-line consolidation. In the CIBMTR study [20], where 59 patients had alloSCT after MAC and 36 after RIC, they reported more relapses with RIC and more TRM with MAC, resulting in similar OS. In our study, the outcome for RIC, including TRM and CIR, appears similar to the outcome for MAC. These results are possibly related to the graft-versus-lymphoma effect and do not favor the use of MAC in this setting. However, due to differences in patients’ characteristics before transplant, the comparison between MAC and RIC might not be relevant despite the use of a propensity score.

Another major unsolved question remains the role of alloSCT as first-line consolidation for eligible patients compared to autoSCT or chemotherapy alone [21]. First, autoSCT has never been prospectively compared to chemotherapy alone in the consolidation of a first-line response for PTCL treatment. A recent matched study based on a propensity score comparing patients managed on an institutional-based strategy of using autoSCT systematically or not did not demonstrate any benefit of autoSCT [9]. However, front-line autoSCT remains a standard treatment in many European countries, according to the European Society of Medical Oncology (ESMO) recommendations [21], and is still recommended in the National Comprehensive Cancer Network (NCCN) guidelines which currently also include the addition of brentuximab in the case of CD30-positive PTCL. The largest prospective autoSCT phase II trial, done on 160 patients by the Nordic Lymphoma Group, reported a 51% OS and 44% PFS [11]. A recent meta-analysis reported a non-negligible TRM of 2 to 6% and a relapse rate of 26 to 36% [22] after autoSCT.

Given the poor prognosis of PTCL, with a cure rate not exceeding 50%, the use of alloSCT in consolidation of a first response (CR1 or PR1) has been recommended and accounts for 48% of the patients in the present study, with a 2- and 4-year OS of 66% and 63%, EFS of 61% and 57%, and CIR of 19% and 20%, respectively. In a monocentric report, alloSCT in CR1/PR1 was systematically used in 49 consecutive PTCL patients [23]. Among these patients, 29 reached CR or PR (60%) and underwent upfront alloSCT with a 2-year OS of 72.5% and a low TRM of 8.2% (RIC, *n* = 24; MAC, *n* = 5). Front-line alloSCT has been prospectively evaluated and compared to autoSCT in two studies. Corradini et al. reported a non-randomized phase II study in 37 patients in response after chemotherapy where 23 received alloSCT and 14 autoSCT. The 4-year PFS was similar for both groups (70% for autoSCT; 69% alloSCT) [24]. The AATT multicentric randomized study compared alloSCT and autoSCT as consolidation in patients achieving SD, PR, or CR after 4 CHOEP cycles. This study was prematurely closed following the interim analysis, and the final results were presented at the ASCO meeting in 2019. In 103 patients (alloSCT arm, *n* = 49; autoSCT arm, *n* = 54), the intent-to-treat analysis failed to demonstrate any significant difference in EFS and OS between arms. As in other studies [11, 12, 25], one third of patients did not reach any consolidation due to early progression. Finally, given donor availability, 41 patients received autoSCT and 26 alloSCT. Despite the absence of late relapse after alloSCT, a significant toxicity responsible for 7 out of 8 deaths prevents to reveal any EFS benefit of this procedure, even when the analysis is limited to patients actually transplanted. For the authors, these results, as well as the possibility of performing salvage alloSCT in second remission, do not favor recommending alloSCT at first remission in PTCL [26].

In accordance, when we evaluated the results from the 116 patients who received alloSCT while in relapse, the 2- and 4-year OS (66% and 61%), EFS (60% and 54%), and CIR (17% and 18%) were similar to those in patients who received alloSCT when in first consolidation (CR1 or PR1), albeit with slightly higher 4-year TRM (30%), compared to the first-line consolidation group (TRM, 24%). The same GVL effect might occur in both setting, but with a higher risk of toxicity in patients receiving alloSCT when in relapse. These data show the necessity to be proactively prepared to perform salvage alloSCT, sometimes early in the disease course, because of the high incidence of first-line failures. Identifying patients with suboptimal response to chemotherapy (e.g., with early PET or other minimal residual disease (MRD) tools) might lead to earlier alloSCT consolidation if the risk of relapse is considered to be important.

The role of alloSCT as consolidation compared to autoSCT has also been challenged in the relapse/refractory setting. AlloSCT is recommended along with autoSCT by the ESMO and NCCN guidelines. In the literature, the largest study reporting outcomes of the relapse/refractory PTCL setting [20] found no difference between alloSCT and autoSCT. However, this study includes a very high number of ALCL (40%), a subtype in which a better prognosis has been demonstrated compared to other PTCL subtypes when an autoSCT strategy is chosen [11], which limits the scope of conclusions to all subtypes. In addition, patients in the alloSCT group had received more treatment lines before transplantation, were less chemosensitive, and had worse remission status at transplantation. The role of alloSCT in other retrospective studies reported encouraging long-term OS post-alloSCT, around 50% [27–33]. With a 30–50% long-lasting survival after alloSCT for relapsed PTCL, the authors of a recent review concluded that alloSCT is a valid option for patients who are eligible, at least after the first relapse, although more prospective studies are also needed in the area of new targeted treatments [34].

One of the major challenges in the treatment of relapsed or refractory PTCL is to induce disease control via bridging therapies to transplantation, especially considering that this population may be underestimated in studies because they do not reach consolidation. In the British Columbia Cancer Agency Lymphoid Cancer database study, only 38 patients (20%) received a transplant, meaning that the rest of the population was not eligible for reasons including age, comorbidities, and lack of tumor control. The 153 non-transplanted patients in this study had a PFS and OS of 3.7 and 6.5 months, respectively [4].

A more recent monocentric study focused on primary refractory patients with a median age of 52 years and shows similar results [35]. Excluding ALCL treated with bentuximab with a high ORR (86%), the ORR of relapse treatment is disappointing, apparently identical whether it is monotherapy or combination.

This overall response rate (ORR) has been well evaluated for monotherapies such as bendamustine (ORR = 55%), gemcitabine (ORR = 51–66%), romidepsine (ORR = 25–61%), or pralatrexate (ORR = 29–43%) [5, 36–39], but with a CR rate usually below 20%. The use of successive treatments leads to an alteration of the performance in status and infectious complications, making the graft even more precarious. Hence, in many centers, patients are transplanted for refractory diseases.

In the present study, 31 patients underwent alloSCT despite having refractory disease at transplantation. The CR rate after alloSCT was 47%, and 55% were still alive after 2 years. Seven of these patients had primary chemo-refractory disease. Three out of the 7 had durable remission, 2 died early, and 2 had PD after alloSCT. Among the 3 responders, 3 developed GvHD. These results suggest that alloSCT can be an option in chemoresistant PTCL.

Our multivariate analysis found an impact of disease status before transplantation, the patients receiving an alloSCT in progressive disease having lower OS than patients transplanted in CR (HR = 2.21, 95% CI 1.25–3.89). This encourages obtaining as much control of the disease as possible before alloSCT. However, considering there is no difference between patients transplanted with CR and those transplanted with PR (HR= 0.72, 95% CI (0.42–1.24)), this should discourage to prolonge salvage treatment before alloSCT with the aim to obtain CR, at the risk of developing comorbidities.

The main limit to this study is the heterogeneity of the population, especially in terms of histologic subtypes. In univariate analysis, no significant difference was found in outcomes comparing histological diagnosis. However, a granular analysis of our data gives some information about the outcomes in PTCL subgroups. For AITL, our results are similar to a large series of 250 patients allotransplanted [40], including 40% in second-line treatment (relapse after autoSCT) and 79% in CR/PR. In this study, Epperla et al. reported a 1-year NRM of 19% and a 4-year PFS, OS, and cumulative incidence of relapse of 49%, 56%, and 21%, respectively.

For the NK/T nasal lymphoma subgroup (*n* = 16), alloSCT was performed mostly (62%) in second-line treatment with a worse OS (2 years OS, 50%) in comparison to the entire group. In a recent article [41] reporting 90 patients with NK/T nasal lymphoma, the outcomes were similar (OS, 34%; relapse rate, 42%; NRM, 30%) without significant difference between patients who had alloSCT in first or second line. These data suggest that subsequent analysis studying the place of alloSCT is needed in patients with this subtype of T lymphomas.

For ALCL, the place of alloSCT has to be redefined, mostly because of the efficient use of brentuximab recently demonstrated in this disease [42]. In our series, the 21 patients with ALK+ ALCL were mostly transplanted in second-line treatment (66%) and had better outcomes compared to the entire cohort with lower TRM, probably resulting from the younger age in this subgroup.

Finally, the recent development of haploidentical SCT offers the possibility to find a suitable donor for the majority of patients [43]. In the near future, in addition to the GVL effect, other strategies (sequential conditioning regimen [44], combination of new drugs such as brentuximab, before [45] or as maintenance after alloSCT [46], could be promising options for these high risk patients, and will have to be prospectively evaluated.

## Conclusion

In this large study, we provide additional data in support of alloSCT to treat PTCL. The relapse rate was rather low, even with RIC, suggesting a strong GVL effect. Nevertheless, toxicity remains a significant issue. While prospective studies ideally should be done before making a firm recommendation for PTCL treatment with alloSCT vs autoSCT, the role of alloSCT appears significant for PTCL treatment, especially in the relapse setting, including for patients with refractory disease. Considering the high rate of front-line treatment failure, eligibility to transplant and donor search should be discussed early with newly diagnosed PTCL patients. As no targeted immune-based therapy is currently available for PTCL, alloSCT may remain the main treatment for aggressive lymphomas.

## Supplementary information

**Additional file 1:.** Patients’ characteristics according to the conditioning regimen (RIC versus MAC)

**Additional file 2:.** Outcomes after post alloSCT relapse (N=65)

**Additional file 3:.** Multivariable analysis for 5-year TRM

## Data Availability

The datasets used and/or analyzed during the current study are available from the corresponding author on reasonable request.

## Abbreviations

SFGM-TC: Société Francophone de Greffe de Moelle et de Thérapie Cellulaire
SCT: Stem cell transplantation
PTCL: Peripheral T cell lymphomas
EFS: Event-free survival
ALCL: Anaplastic large cell lymphoma
OS: Overall survival
PFS: Progression-free survival
CAR: Chimeric antigen receptor
GVL: Graft-versus-lymphoma
RIC: Reduced-intensity conditioning
DLI: Donor lymphocyte infusion
GvHD: Graft versus host disease
TRM: Therapy-related mortality
CR: Complete response
PR: Partial response
PD: Progressive disease
CIR: Cumulative incidence of relapse
MAC: Myeloablative conditioning
NOS: Not otherwise specified
AITL: Angioimmunoblastic T lymphoma
ALK+/−: With/without anaplastic lymphoma kinase mutation
ATLL: Adult T cell leukemia/lymphoma
HSTL: Hepatosplenic T cell lymphoma
EATL: Enteropathy-associated T cell lymphoma
T-LGL: T large granular lymphocytic leukemia
ESMO: European Society of Medical Oncology
NCCN: National Comprehensive Cancer Network
SD: Stable disease
MRD: Minimal residual disease
PET: Positron emission tomography
ORR: Overall response rate
