# Supplementary material for: Allogeneic stem cell transplantation for peripheral T cell lymphomas: a retrospective study in 285 patients from the Société Francophone de Greffe de Moelle et de Thérapie Cellulaire (SFGM-TC)
Source: J Hematol Oncol. 2020 May 19;13:56. doi: 10.1186/s13045-020-00892-4 (PMC7236365; doi:10.1186/s13045-020-00892-4)
Supplement: Supplementary file 1 — Additional file 1:. Patients’ characteristics according to the conditioning regimen (RIC versus MAC) [file 13045_2020_892_MOESM1_ESM.pdf]

<u>Additional file 1</u>:
Patients' characteristics according to the conditioning regimen (RIC versus MAC).

		RIC (med, %)	MAC (med, %)	*P*
N		174	107	
Age at transplant (yo)		54	40	<0.0001
Nb lines of treatment > 2		40%	28%	0.077
Previous auto-SCT		44%	16%	<0.001
Disease status at transplant	CR	67%	55%	0.015
	PR	21%	37%	
	PD	12%	8%	
Median time from diagnosis to allo-SCT (months)		15	10	0.0003

Legend: RIC: reduced-intensity conditioning regimen; MAC: myeloablative conditioning regimen; med: median; yo: years old; nb: number; SCT: Stem cell transplantation; CR: complete remission; PR: partial remission; PD: progressive disease.
