# Supplementary material for: Allogeneic stem cell transplantation for peripheral T cell lymphomas: a retrospective study in 285 patients from the Société Francophone de Greffe de Moelle et de Thérapie Cellulaire (SFGM-TC)
Source: J Hematol Oncol. 2020 May 19;13:56. doi: 10.1186/s13045-020-00892-4 (PMC7236365; doi:10.1186/s13045-020-00892-4)
Supplement: Supplementary file 2 — Additional file 2:. Outcomes after post alloSCT relapse (N=65) [file 13045_2020_892_MOESM2_ESM.pdf]

Additional Files 2

Outcomes after post alloSCT relapse (N=65)

	**N (%)**	**Status at last follow up**
No treatment at relapse	29 (45%)	Dead from relapse: 28/29<br>Dead in CR: 1/20 (CR after withdrawal of immune suppression drugs)
Chemotherapy or radiotherapy alone	23 (35%)	4/23 alive and in CR
Chemotherapy + DLI	4 (6%)	5/9 alive and in CR
DLI alone	5 (8%)	
Unknown	4	-

Legend: SCT: Stem cell transplantation; CR: complete remission; DLI: donor lymphocyte infusion.
