# Supplementary material for: Allogeneic stem cell transplantation for peripheral T cell lymphomas: a retrospective study in 285 patients from the Société Francophone de Greffe de Moelle et de Thérapie Cellulaire (SFGM-TC)
Source: J Hematol Oncol. 2020 May 19;13:56. doi: 10.1186/s13045-020-00892-4 (PMC7236365; doi:10.1186/s13045-020-00892-4)
Supplement: Supplementary file 3 — Additional file 3:. Multivariable analysis for 5-year TRM [file 13045_2020_892_MOESM3_ESM.pdf]

Additional file 3-

Multivariable analysis for 5-year TRM:

**Multivariable analysis for 5year TRM**			
		**HR (95%CI)**	***p***
Age at transplant		1.02 (1.00-1.05)	0.052
Karnofsky score (%)	90-80 vs 100	1.83 (0.96-3.46)	0.065
	<80 vs 100	3.43 (1.09-10.7)	0.034
Sex mismatch D/R	Female/Male *vs* others	1.59 (0.92-2.77)	0.096
Number of lines of treatment	⩽ 2	0.59 (0.35-0.99)	0.047

Legend: TRM: toxic-related mortality; HR: hazard ratio; D: donor; R: recipient.
